# PERSONAL STRATEGIES FOR MANAGING EVERYDAY ACTIVITY CHALLENGES AMONG ADULTS AGING WITH VISION LOSS

**DOI:** 10.1093/geroni/igac059.3124

**Published:** 2022-12-20

**Authors:** Kara Mumma, Elena Remillard, Wendy Rogers

**Affiliations:** Georgia Institute of Technology, Atlanta, Georgia, United States; Georgia Institute of Technology, Atlanta, Georgia, United States; University of Illinois Urbana Champaign, Champaign, Illinois, United States

## Abstract

An estimated 6% of adults in the United States ages 65 and older are living with vision loss and may experience other age-related declines and conditions co-morbidly (e.g., arthritis, hypertension, hearing loss). As such, they are likely to experience challenges in performing everyday activities, but little is known about their personal strategies or innovative solutions for managing activity challenges. The Aging Concerns, Challenges, and Everyday Solution Strategies (ACCESS) study explored everyday activity challenges and strategies among older adults (Nf60; ages 60–79) with long-term vision loss (prior to age 50). The current analysis focused on understanding participants’ own methods for managing activity challenges, or things they have come up with, beyond traditional methods, such as getting help from other people (e.g., caregiver) or using assistive devices (e.g., white cane, screen reader). We coded response strategies among participants’ own methods, and the most frequently reported methods included: learning/familiarity/organization, planning ahead/prioritizing, and perseverance/patience/assertiveness. Response codes were classified using the Selection, Optimization, and Compensation model to better understand how these strategies are being used to adapt to losses. The most common strategies were classified as elective selection with optimization, which are strategies that entail continued performance of challenging activities through behavioral methods, without bringing in any new means to assist. Illustrative example quotes are featured to provide insight on the context of personal strategies. With greater understanding of solutions people aging with vision loss are employing to manage activity challenges, we can increase awareness about effective solutions and identify opportunities for supportive technology solutions.

